# Supplementary material for: Identification and functional analysis of non-coding regulatory small RNA FenSr3 in Bacillus amyloliquefaciens LPB-18
Source: PeerJ. 2023 May 15;11:e15236. doi: 10.7717/peerj.15236 (PMC10194069; doi:10.7717/peerj.15236)
Supplement: Supplemental Information 4 [file peerj-11-15236-s004.zip › KO/CK-vs-T1_map/map04931.html]

KEGG PATHWAY: Insulin resistance - Reference pathway


|  |  |
| --- | --- |
| **Insulin resistance - Reference pathway** |  |

[
Pathway menu
| Organism menu
| Pathway entry
| Show description
| User data mapping
]

Insulin resistance is a condition where cells become resistant to the effects of insulin. It is often found in people with health disorders, including obesity, type 2 diabetes mellitus, non-alcoholic fatty liver disease, and cardiovascular diseases. In this diagram multiple mechanisms underlying insulin resistance are shown: (a) increased phosphorylation of IRS (insulin receptor substrate) protein through serine/threonine kinases, such as JNK1 and IKKB, and protein kinase C, (b) increased IRS-1 proteasome degradation via mTOR signaling pathway, (c) decreased activation of signaling molecules including PI3K and AKT, (d) increase in activity of phosphatases including PTPs, PTEN, and PP2A. Regulatory actions such as oxidative stress, mitochondrial dysfunction, accumulation of intracellular lipid derivatives (diacylglycrol and ceramides), and inflammation (via IL-6 and TNFA) contribute to these mechanisms. Consequently, insulin resistance causes reduced GLUT4 translocation, resulting in glucose takeup and glycogen synthesis in skeletal muscle as well as increased hepatic gluconeogenesis and decreased glycogen synthesis in liver. At the bottom of the diagram, interplay between O-GlcNAcylation and serine/threonine phosphorylation is shown. Studies suggested that elevated O-GlcNAc level was correlated to high glucose-induced insulin resistance. Donor UDP-GlcNAc is induced through hexosamine biosynthesis pathway and added to proteins by O-GlcNAc transferase. Elevation of O-GlcNAc modification alters phosphorylation and function of key insulin signaling proteins including IRS-1, PI3K, PDK1, Akt and other transcription factor and cofactors, resulting in the attenuation of insulin signaling cascade.

|  |  |  |
| --- | --- | --- |
| Reference pathway | 184% 150% 122% 100% 82% 67% 55% | 图片下载 |
